# Assessment of Fish Biomass and Distribution in a Nuclear Power Plant’s Water Intake Zone Using Acoustic and Trawl Methods

**DOI:** 10.3390/ani15070987

**Published:** 2025-03-29

**Authors:** Zuli Wu, Yunpeng Song, Guoqing Zhao, Yongchuang Shi, Yumei Wu, Shengmao Zhang

**Affiliations:** 1 Key Laboratory of Fisheries Remote Sensing, Ministry of Agriculture and Rural Affairs, East China Sea Fisheries Research Institute, Chinese Academy of Fishery Sciences, Shanghai 200090, China; wuzl@ecsf.ac.cn (Z.W.); syp9805@163.com (Y.S.); zgq617717@163.com (G.Z.); shiyc@ecsf.ac.cn (Y.S.); 2 School of Information Engineering, Huzhou University, Huzhou 313000, China; 3 College of Marine Living Resource Sciences and Management, Shanghai Ocean University, Shanghai 201306, China

**Keywords:** resource density, acoustic assessment, dominant species, spatiotemporal distribution

## Abstract

The cooling water intake systems of coastal nuclear power plants are frequently clogged by marine organisms in the water intake area. This study uses hydroacoustic (BioSonics DT-X, 199 kHz) and trawling methods (20 mm codend mesh size) to research fish spatiotemporal dynamics. Species composition, seasonal variations in fish abundance, and target strength were analyzed. Pearson’s correlation analysis revealed a significant negative correlation between water depth and resource density. Significant differences in fish abundance and biomass density were observed among seasons and stations. An analysis of dominant species showed that the IRI (index of relative importance) of *Collichthys lucidus* was highest in February 2023 (5736.54), while *Harpadon nehereus* had the highest IRI in November 2023 (2309.17). The distribution and abundance estimates of dominant species from acoustic surveys and biological trawling were highly consistent, demonstrating the applicability and reliability of hydroacoustic methods in fish resource assessments. Through the identification of dominant species as well as the distribution patterns of fish within the water intake area, valuable data support can be provided for the confirmation of those organisms that are prone to clogging the cooling water source. Moreover, it lays a solid foundation for the development of relevant protection work.

## Simple Summary

The cooling water intake system of coastal nuclear power plants is vulnerable to fluctuations in fish resources, which may lead to problems, such as clogging of the cooling system. To solve this problem, this study adopted a combined method of acoustic technology and trawling to conduct an in-depth investigation of fish populations in the water intake area of a nuclear power plant in Fujian. This study revealed that the species composition and abundance of fish underwent substantial variations across different seasons and locations. Notably, certain dominant fish species, including *Collichthys lucidus* and *Harpadon nehereus*, displayed distinct levels of importance in diverse months. Additionally, the results clearly indicated that the fish tended to concentrate in shallow waters. This research further demonstrated the reliability of acoustic methods in the assessment of fish resources. By identifying the dominant species and the distribution of fish in the water intake area, it can provide data support for the confirmation of organisms that are likely to clog the cooling water source and also lay a foundation for the development of relevant protection work.

## 1. Introduction

Marine organisms, floating debris, and sea ice entering the cooling system during the water intake process of nuclear power plants can clog filtration devices, making water intake difficult for pump units and threatening the safe and stable operation of the plant [1,2,3,4]. From 1996 to 2021, there were at least 108 clogging incidents in the cold source water intake systems of nuclear power plants worldwide, among which 63.8% were caused by the clogging of marine organisms. In recent years, marine organisms, such as *Acaudina molpadioides*, microalgae, and aggregating fish species, have frequently blocked cooling water intake systems, significantly disrupting the normal operation of nuclear units [2,5,6,7]. To address the nuclear safety risks posed by these biological outbreaks, it is essential to conduct detailed studies on the abundance and spatiotemporal distribution of dominant biological populations within the water intake zones of nuclear power plants [8]. Accurately grasping the quantity and spatiotemporal distribution of dominant biological populations in the water intake areas of nuclear power plants is of crucial importance for identifying, monitoring, and predicting the marine organisms that may affect the cooling systems and ensuring the safe operation of nuclear power units [9,10].

Acoustic technology has demonstrated significant technical advantages in both the identification of terrestrial, subterranean [11,12], and marine biological populations and the assessment of their abundance [13,14,15,16]. This technology emits sound waves vertically into the water using scientific echo sounders and analyzes the returned echo signals to determine the distribution and biomass of aquatic organisms, meeting the demand for efficient and large-scale surveys [15]. High-resolution acoustic imaging allows for a precise analysis of the distribution density and echo characteristics of aquatic populations, providing deeper insights into aquatic ecosystems [17]. Chinese researchers have employed acoustic technology to monitor and assess the resources of *Collichthys lucidus*, offering scientific support for the safe operation of cooling water intake at nuclear power plants [5]. In addition, digital omnidirectional sonar and dual-beam acoustic systems have been used to estimate the biomass of individual fish schools, with successful applications in assessing fish populations, such as *Clupea pallasi* and *Pneumatophorus japonicus* [15,18,19]. In addition, through acoustic surveys, combined with the analysis of the reproductive and growth habits of target species, it is possible to identify the migration and aggregation characteristics of risk species, such as *Collichthys lucidus*, and provide a basis for formulating effective mitigation strategies [5]. However, the behavioral and echo characteristics of fish vary underwater, and marine environmental conditions are influenced by seasonal and geographic factors, complicating acoustic monitoring [20]. As such, nuclear power plants need to tailor acoustic monitoring methods and parameters according to specific environmental conditions and the characteristics of target species [21]. Despite its advantages, acoustic technology still faces certain challenges. Complex underwater environments can introduce strong speckle noise, blurred boundaries, and weak texture information, affecting sonar imaging quality [22,23,24]. During acoustic assessments, the survey is often terminated at a certain height above the seabed to eliminate bottom-reflected signals, making it unsuitable for accurately assessing benthic fish species [25,26]. Therefore, acoustic surveys mainly target non-benthic fish, such as those in the Gobiidae, Sciaenidae, and Cynoglossidae families, etc.

During the water intake process of nuclear power plants, the entry of marine organisms into the cooling system may lead to blockages, threatening the safe and stable operation of the nuclear power plants. Therefore, it is of vital importance to study the quantity and spatiotemporal distribution of dominant biological populations in the water intake areas. Currently, further research is still needed on how to comprehensively utilize multiple methods to accurately and comprehensively assess fish resources in the water intake areas of nuclear power plants and how to deeply understand the influence mechanism of environmental factors on fish resources. In March 2021, February 2023, and November 2023, biological bottom trawl surveys and sonar surveys were simultaneously carried out in the cooling water intake area of a nuclear power plant in the Fujian Province. By integrating the sonar data and the data of marine organisms captured by trawl nets, a comprehensive analysis was conducted on the composition and spatiotemporal distribution of fish resources. The main contributions of this study are as follows: 1. Innovatively combining acoustic and trawl methods, we conducted systematic multi-year, multi-season investigations in the nuclear power plant’s water intake zone. This study rigorously revealed spatiotemporal variations in fish species composition and abundance, yielding a robust dataset for ecological assessments. 2. Precisely tracking seasonal shifts in dominant fish species dynamics, this study unveiled their adaptive strategies to environmental changes. 3. This study validated the reliability of acoustic methodologies for fish stock assessment, establishing critical technical references for related research, while providing scientifically robust safeguards for water intake system safety management in nuclear power plants.

## 2. Materials and Methods

### 2.1. Survey Area and Station Design

**Figure 1:**
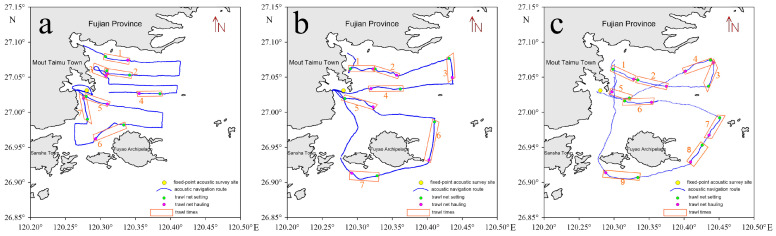
Study area, acoustic survey routes, and distribution of trawl stations. (Cruise: (**a**) March 2021; (**b**) February 2023; (**c**) November 2023).

Fish resource surveys were conducted in the waters within a 15-kilometer radius of the nuclear power plant intake from 12 to 15 March 2021 (winter), 7 to 8 February 2023 (winter), and 20 to 21 November 2023 (autumn). The survey routes were planned following the requirements of the Specifications for Oceanographic Survey—Part 6: Marine Biological Survey (GB/T 12763.6-2007) while also taking into account the water depth, distribution of aquaculture facilities, and drift gillnets in the area [27]. To comprehensively assess fish resources in the target waters, four acoustic transects were established. The cumulative transect distances covered during the surveys were 59.4 n miles (March 2021), 50.92 n miles (February 2023), and 54.04 n miles (November 2023). In addition, seven biological trawl stations were set up for each survey (with nine stations in the November 2023 survey), and trawl stations were placed along each acoustic transect. Table 1 and Figure 1 present the geographical locations of the survey stations and details of the trawl operations for each survey.

### 2.2. Biological Sample Data Analysis

The analysis of dominant catches was conducted using the index of relative importance (IRI) proposed by Perpetua et al. [28]:

IRI = (N + W) × F(1)

In Equation (1), N represents the percentage of the number of individuals of a species relative to the total catch; W represents the percentage of the biomass of a species relative to the total catch; and F represents the frequency of occurrence of the species. Species with an IRI ≥ 1000 are classified as dominant species, while those with 100 ≤ IRI < 1000 are considered key species.

The estimation of trawl resource density at each survey station was conducted using the swept-area method. Fish resource density was calculated based on the catch (in both biomass and number of individuals) and the area swept by the trawl net at each station [29]. The calculation formula is as follows:

(2) $$\rho_{i}=\frac{C_{j}}{a_{i}q}$$

In Equation (2), *p_i_* represents the resource density at station *i* (in terms of biomass: kg/km^2^; or abundance: ind./km^2^); *C_i_* is the catch per hour at station *i* (in terms of biomass: kg/h; or abundance: ind./h); *a_i_* is the swept area per hour at station *i* (km^2^/h), calculated as the product of the horizontal net opening width (km) and the towing distance (km) divided by the actual towing time (h). The towing distance is the product of the towing speed (km/h) and the actual towing time (h). *q* represents the catchability coefficient of the fishing gear, with species-specific catchability coefficients listed in Table 2.

### 2.3. Acoustic Survey Data Collection

Acoustic data and navigation data were dynamically collected using a BioSonics DT-X scientific echosounder (frequency: 199 kHz, High Falls, NY, USA) and an external Garmin GPS60CSx GPS device (Olathe, KS, USA). The entire instrument was firmly installed (with an inclination angle of <2°), and it remained stable during the underway survey to ensure the quality of the acoustic survey data. Before the survey, the gain coefficients for both the transmission and reception of the echosounder system were calibrated on-site using the standard target method with copper spheres of 60.0 mm, 32.1 mm, and 23.0 mm in diameter, following international standards. Before starting the survey, the environmental parameters, such as temperature and salinity, were configured in the echosounder. The detection range was set based on the depth, topography, and geomorphology of the target waters. Set the parameters of environmental factors, such as the temperature (16.5 °C) and salinity (28.0 ppt), inside the equipment. Based on the water depth of the target sea area (with an average of 19.1 m), set the vertical detection range to 0.5–40 m (truncated at 0.3 m above the seabed) to configure the detection range. The digital pulse repetition rate was set to 4 pings per second. During the transect survey, the vessel Minfudingyu 02786 maintained a cruising speed of 3 to 4 knots.

### 2.4. Acoustic Survey Data Analysis

The analysis of the acoustic data was conducted using the Echoview 8.2 fisheries acoustic data processing software. The collected acoustic echo image data were processed using the echo integration method, during which background noise signals unrelated to the target species were eliminated, including sea surface noise, zooplankton noise, mechanical disturbance noise, and multiple bottom echo noise. The analysis of the acoustic data focused on the water column extending from 1.0 m below the transducer surface to 0.3 m above the seabed. All echo images from the survey transects were carefully examined, and manual editing was performed as necessary to regenerate the bathymetric maps. To exclude echo signals from weak scatterers, such as zooplankton, the minimum thresholds for volume backscattering strength and target strength in the echo images were set at −60 dB and −55 dB, respectively, with the basic integration range unit set at 0.5 n mile. After the parameter settings were completed, automatic classification and counting were conducted using a trajectory tracking analysis combined with trajectory tracking technology to calculate fish density in the surveyed water volume [33].

Statistical analyses were performed on the quantity, length, and weight distribution of the catch during the trawling period. Additionally, acoustic fishery resource data from the trawling period were extracted to analyze the distribution of resource abundance and target strength within the acoustic data. The composition of the measured trawl catch and its length-weight distribution were compared to validate the consistency between acoustic data and biological trawl survey results.

Following the principles and procedures of various multi-species marine fishery resource acoustic assessment methods, a fish resource assessment was conducted, with acoustic processing integrated against the trawl catch data. The abundance density of each fish species in the surveyed area was calculated as follows [34]:

(3) $$\rho_{i}=C_{j}\times \frac{NASC}{4\pi \sigma }$$

In Equation (3), *C_j_* represents the percentage of the total biomass occupied by the given fish species *j* in the analyzed area. *NASC* (m^2^·n mile^2^, Nautical Area Scattering Coefficient) is the total integrated value allocated to biological species at the site transect, and σ is the average acoustic cross-section (m^2^, backscattering cross-section) of all biological species in the analyzed area, where

(4) $$\sigma =\sum_{j=1}^{n}\left(C_{j}\times 10^{\frac{{TS}_{j}}{10}}\right)$$

In Equation (4), *n* represents the total number of fish species in the analyzed area, and *TS*_*j*_ denotes the target strength (dB) of the given fish species *j* in the analyzed area, which can be expressed as follows:

(5) $${TS}_{j}=20\log l_{j}+b_{20}$$

In Equation (5), *l_j_* represents the average length (cm) of the fish species *j*, and *b*_20_ is the target strength parameter (dB) for species *j*, obtained from the existing literature. The *b*_20_ coefficients for certain marine fish families commonly found in the study area are listed in Table 2. The resource density of fish species *j* can be expressed as follows:

(6) $$p_{j}=C_{j}\times \frac{NASC}{4\pi \sigma }\times \frac{W_{j}}{1000}$$

In Equation (6), *p_j_* is expressed in kg·n mile^−2^, and *W_j_* represents the average body mass (g) of fish species *j* in the analyzed area. The resource density and its distribution along the transect are calculated to evaluate the existing fishery resource biomass in the area as follows:

(7) $$W_{T}=\sum_{j=1}^{k}A_{j}\sum_{j=1}^{n}p_{j}$$

In Equation (7), *k* represents the number of different fish species whose resource abundance is being studied in the analyzed area, *n* is the total number of fish species in the area, *A_j_* denotes the resource density (kg·n mile^−2^) of fish species *j* in the analyzed area, and *p_j_* is the resource density (kg·n mile^−2^) of fish species *j*.

## 3. Results

### 3.1. Fish Resource Trawl Survey

#### 3.1.1. Spatiotemporal Distribution of Fish Resource Abundance Density

A total of 54 species of swimming animals were identified during the three surveys (March 2021), 43 species (February 2023), and 45 species (November 2023). Among these, the number of fish species was 26, 29, and 27, accounting for 48.15%, 67.44%, and 60% of the total swimming animal species, respectively (Table 3). The average fish resource density during the February 2023 survey was the highest (17,267 ind./km^2^), while the lowest was observed in March 2021 (6855 ind./km^2^). The average fish weight density was highest in November 2023 (198.659 kg/km^2^) and lowest in February 2023 (19.022 kg/km^2^) (Table 3).

**Figure 2:**
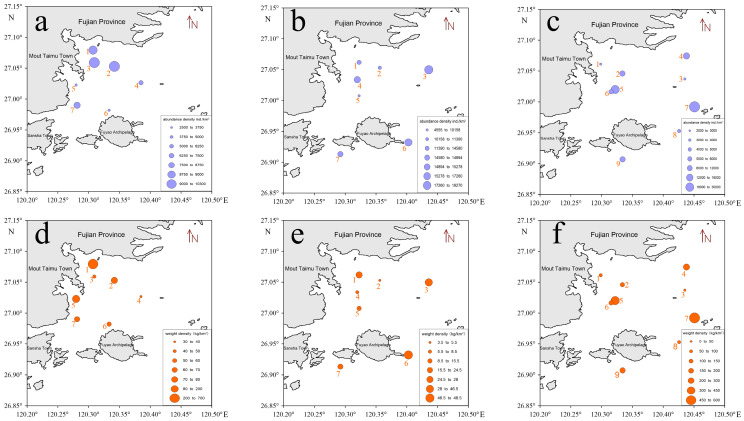
Distribution of fish resource density and biomass density in the survey area. (**a**–**c**) represent the fish resource density distribution at each station during the cruises in March 2021, February 2023, and November 2023, respectively; (**d**–**f**) represent the fish biomass density distribution at each station during the cruises in March 2021, February 2023, and November 2023, respectively.

A one-way ANOVA showed significant differences in resource density and weight density across the survey stations during all three investigations (Figure 2 and Table 4). In March 2021, the spatial distribution differences in resource density and weight density were significant (F = 2.567, *p* < 0.05; F = 5.773, *p* < 0.01). In February 2023, significant differences were also noted in resource density and weight density (F = 2.661, *p* < 0.05; F = 2.070, *p* < 0.05). The differences in resource density and weight density in November 2023 were even more pronounced (F = 9.378, *p* < 0.05; F = 2.121, *p* < 0.05).

#### 3.1.2. Dominant Species Composition

**Figure 3:**
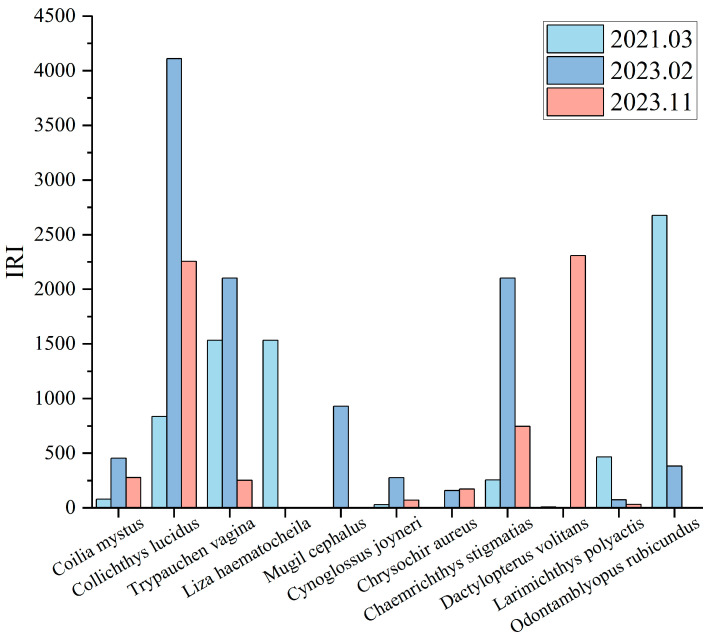
Relative importance index (IRI) of major catches from fish trawl surveys conducted in March 2021, February 2023, and November 2023.

Based on the Equation (1) for the relative importance index (IRI) of dominant catches, species with an IRI greater than 1000 were identified as dominant fish species, as shown in Figure 3. In the March 2021 fish resource survey, three dominant catches were identified as follows: *Odontamblyopus rubicundus*, *Trypauchen vagina*, and *Liza haematocheila*, with IRIs of 2678.04, 1534.93, and 1534.40, respectively. In the February 2023 survey, four dominant catches were recorded, with the highest IRI belonging to *Collichthys lucidus* at 5736.54, followed by *Chaemrichthys stigmatias*, *Trypauchen vagina*, and *Mugil cephalus*, with IRIs of 3191.98, 2136.69, and 1093.18, respectively. In the November 2023 survey, two dominant catches were noted, with the highest IRI for *Dactylopterus volitans* at 2309.17 and *Collichthys lucidus* at 2256.28.

### 3.2. Acoustic Survey Investigation

#### 3.2.1. Distribution of Fish Target Strength and Body Length

**Figure 4:**
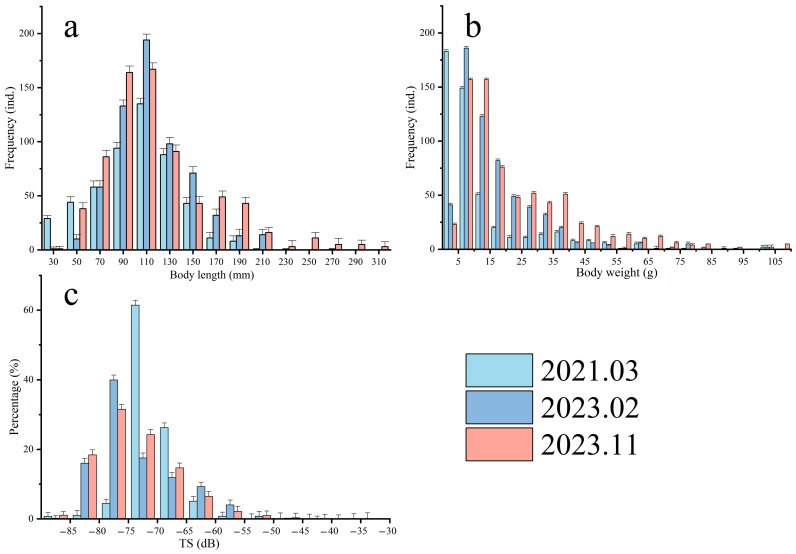
Distribution of fish body length (**a**), body weight (**b**), and acoustic survey target strength (**c**) in the trawl surveys conducted in March 2021, February 2023, and November 2023 (mean ± SD).

During the surveys conducted in March 2021, February 2023, and November 2023, a total of 689 fish (with a total weight of 33,556.5 g), 638 fish (with a total weight of 32,350.8 g), and 625 fish (with a total weight of 30,953.6 g) were collected, respectively. The distribution of fish body length and weight for each survey is shown in Figure 4a,b. According to the average body length and weight distribution results from the three surveys, the fish samples from March 2021 had the smallest body length, with 99.8% of the samples measuring less than 100 mm and an average length of 12.70 ± 15.88 mm. At the same time, 95.1% of the samples weighed less than 100 g, with an average weight of 54.70 ± 168.99 g. In contrast, the November 2023 survey exhibited larger fish in both body length and weight, with 70.07% of the samples falling within the range of 70 to 130 mm and weights ranging from 5 to 35 g, accounting for 80.77% of the total samples. The results from the February 2023 survey indicated that the fish body length and weight were intermediate between the two previous surveys, with 95.3% of the samples weighing less than 100 g and an average body length of 124.03 ± 66.06 mm. Overall, the fish resources captured during the trawl surveys predominantly consisted of small- to medium-sized individuals (body length of 70–130 mm, weight of 5–35 g), indicating a significant prevalence of this size category in the surveyed waters.

In general, the target strength (TS) of fish is closely related to individual size: larger body sizes are associated with greater capability to reflect sound waves, resulting in higher target strength, and vice versa [35]. In this study, based on the echogram data from the simultaneous acoustic surveys during trawling and the statistical analysis from the trawl surveys, we obtained the target strength distribution of dominant fish species for each survey (Figure 4c). According to the acoustic detection analysis based on Equation (5), the TS range for dominant populations across different survey trips is shown in Table 5. In the March 2021 survey, the dominant species were the *Odontamblyopus rubicundus* and *Trypauchen vagina*, together accounting for 66.30% of the total individual count from the trawl survey. The target strength of these goby species ranged from −75.91 to −66.32 dB, corresponding to 61.42% of the individuals counted in the acoustic survey. The February 2023 survey revealed that *Collichthys lucidus*, goby species, and *Mugil cephalus* were the primary species, collectively comprising 95.82% of the total trawl sample count. Among them, the target strength range for *Collichthys lucidus* was from −53 to −43 dB, while the goby species ranged from −57 to −47 dB. In the November 2023 autumn survey, the dominant species in terms of numbers were *Dactylopterus volitans*, *Collichthys lucidus*, and *Chaemrichthys stigmatias*, with trawl data indicating that the relationship between these fish body lengths and target strengths fell within the range of −60 to −50 dB. In the acoustic survey, the proportion of biological individuals within this target strength range was 3.47%.

#### 3.2.2. Acoustic Assessment of Fish Resources

**Figure 5:**
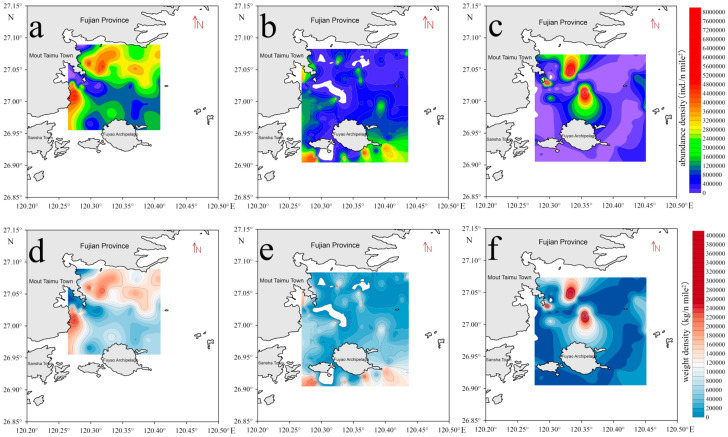
Distribution of fish resource density and biomass density from acoustic surveys. (**a**–**c**) represent the fish resource density distribution during the cruises in March 2021, February 2023, and November 2023, respectively; (**d**–**f**) represent the fish biomass density distribution during the cruises in March 2021, February 2023, and November 2023, respectively.

During the analysis of acoustic data, echoes affected by net operations and surface bubbles due to wind and waves were removed. Subsequently, regional and integral cells were created, and the echo integration values, maximum target strength, average target strength, and minimum target strength of the fish in the study area were exported (Table 6). Based on the fish density calculation Equations (3), (6), and (7), combined with the habitat density and biomass calculation results from the acoustic survey data (Figure 5). In the spring survey conducted in March 2021, the average numerical density in the study area was 1.23 × 10^5^ ind./n mile^2^, with a maximum of 2.98 × 10^5^ ind./n mile^2^ and a minimum of 1.48 × 10^4^ ind./n mile^2^. The average biomass density was 9.29 × 10^4^ kg/n mile^2^, with a maximum of 2.26 × 10^5^ kg/n mile^2^ and a minimum of 1.12 × 10^4^ kg/n mile^2^. In the spring acoustic survey conducted in February 2023, considering the area and average depth of the survey site, the average fish density was 2.78 × 10^5^ ind./n mile^2^, with a maximum of 5.96 × 10^6^ ind./n mile^2^ and a minimum of 120.07 ind./n mile^2^. The average biomass density was 1.41 × 10^4^ kg/n mile^2^, with a maximum of 3.02 × 10^5^ kg/n mile^2^ and a minimum of 6.09 kg/n mile^2^. In the autumn survey conducted in November 2023, the average fish density was calculated to be 5.48 × 10^5^ ind./n mile^2^, with a maximum of 7.90 × 10^6^ ind./n mile^2^ and a minimum of 2.48 × 10^4^ ind./n mile^2^. The average biomass density was 1.26 × 10^4^ kg/n mile^2^, with a maximum of 4.01 × 10^5^ kg/n mile^2^ and a minimum of 1257.77 kg/n mile^2^.

#### 3.2.3. Influence of Water Depth on Fish Resource Density

**Figure 6:**
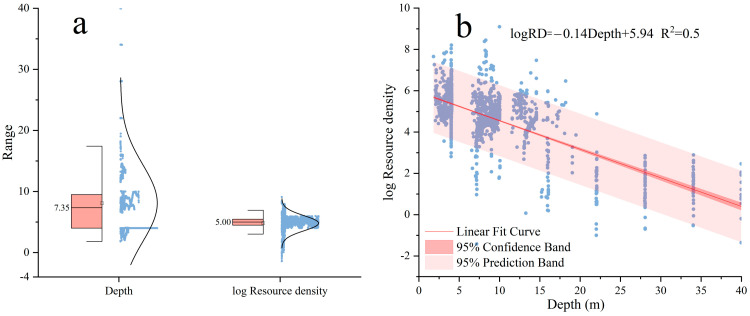
Water depth and log resource density: (**a**) boxplot with normal distribution and (**b**) scatter plot with linear fit.

To explore the impact of water depth on fish resource density, this study integrated acoustic survey data from March 2021, February 2023, and November 2023 to conduct a comprehensive analysis of fish resource density at different depths. A normality test (Figure 6a) revealed that the log-transformed data of water depth and fish resource density followed a normal distribution, meeting the requirements for the correlation analysis. Pearson’s correlation analysis was subsequently employed to evaluate the relationship between water depth and fish resource density. The results showed a significant negative correlation between water depth and fish resource density (r = −0.769, *p* < 0.01). This strong negative correlation indicates that fish resource density decreases markedly with increasing water depth. Specifically, fish resources were more concentrated in shallower areas (approximately within the 3–17 m depth range), accounting for 94.14% of the total density. In contrast, resource density declined rapidly in deeper waters (Figure 6b). The highest log-transformed fish resource density in shallow areas was logRD = 9.096, whereas in deeper areas exceeding 17 m, the log-transformed density dropped to its lowest value, approximately logRD = −1.421.

## 4. Discussion

### 4.1. Spatiotemporal Distribution of Species

The results from the three surveys indicate significant variations in the number of fish species and resource density across different sites, months, and years. The observed spatiotemporal variations in fish species composition and resource density across different sites, months, and years can be attributed to a combination of seasonal ecological behaviors, environmental drivers, and species-specific adaptations [36,37]. A total of 54, 43, and 45 swimming species were identified, with 26, 29, and 27 fish species identified in each survey, respectively, indicating a certain level of biodiversity. These fluctuations in species numbers reflect the seasonal dynamics of the ecosystem [38,39,40]. The February 2023 survey recorded the highest number of fish species, along with the highest individual density of 17,267 ind./km^2^, suggesting favorable environmental conditions for the occurrence and distribution of certain fish populations during this season. Dominant species observed included *C. lucidus*, *C. stigmatias*, *T. vagina*, and *M. cephalus*. The decrease in precipitation in winter may lead to higher salinity, which could attract more euryhaline marine species to enter shallow water areas, thereby increasing the species diversity and the resource quantity in this region [41]. In addition, species like *C. lucidus* usually have a higher abundance in spring and summer. However, if the water temperature is relatively high in winter (for example, close to its suitable spawning temperature of 14–17 °C), its active period may be prolonged, resulting in it still maintaining a high abundance in winter [42,43]. In addition, we analyzed that the possible reason for the relatively high water temperature in the nuclear area may be due to the warm water discharge from the power station.

The results of the one-way analysis of variance (ANOVA) indicate significant spatial differences in resource density and biomass density across different sites during the three surveys. These differences are likely closely related to the influence of water depth on the environmental conditions [44]. This was further confirmed by the analysis of acoustic survey data [41,45,46], which revealed a significant negative correlation between water depth and fish resource density, with the resource density decreasing as the depth increases (r = −0.769, *p* < 0.01). This finding aligns with biological and ecological expectations, as shallow waters typically exhibit higher primary productivity and abundant food resources, attracting large fish aggregations [37,47]. Additionally, shallow areas tend to have higher temperatures, providing favorable conditions for the growth and reproduction of many fish species and promoting the concentration of fish populations in these areas [48,49]. As a result, shallow waters serve as hotspots for fish resources in the surveyed region. Overall, this study reveals that water depth is a critical environmental factor influencing fish resource density, particularly in coastal ecosystems, such as the water intake zone of a nuclear power plant. The findings provide scientific evidence for understanding the impact of water depth on fish distribution and offer valuable insights for the management and conservation of coastal ecosystems.

### 4.2. Diversity and Variation Analysis of Dominant Species

In the March 2021 survey, three dominant fish species were identified as follows: *O. rubicundus*, *A. hexanema*, and *M. cephalus*. In the February 2023 survey, four dominant species were recorded as follows: *C. lucidus*, *C. beniteguri*, *T. vagina*, and *M. cephalus*. Notably, *T. vagina* was identified as a dominant species in both surveys, indicating a stable population and ecological advantage during the spring season. This stability is likely associated with favorable spring conditions, including optimal water temperature, nutrient concentrations, and abundant benthic food resources. Previous studies suggest that the optimal growth temperature and egg-hatching temperature for *T. vagina* range from 14.6 °C to 16.7 °C and 14 °C to 17 °C, respectively [50]. These conditions align well with the environmental factors present in early spring in the coastal waters near Ningde. *C. lucidus* emerged as a dominant species in both the February and November 2023 surveys, with its IRI (index of relative importance) peaking at 5736.54 in February and reaching a secondary high of 2256.28 in November. This species demonstrates strong adaptability to seasonal changes, thriving across a wide range of temperatures and salinities as a benthopelagic fish. Its ability to secure adequate food resources and habitat conditions in different seasons reflects its resilience and reproductive capability [36,42,51]. However, in the November 2023 survey, only two dominant species were identified as follows: *H. nehereus* and *C. lucidus*. The reduced number of dominant species and the high IRI of *H. nehereus* (2309.17) suggest that it became a primary species in autumn, likely due to its high mobility and broad dietary range. Studies on the feeding habits of *H. nehereus* indicate that its feeding intensity varies seasonally, with the highest average stomach fullness index occurring in autumn and the lowest in spring [52,53]. These variations highlight the fish community’s responses to seasonal environmental changes, such as shifts in water temperature, nutrient supply, and light conditions. These factors play a crucial role in shaping ecological niches and driving population dynamics among fish species.

### 4.3. Acoustic Evaluation of Fish Target Strength and Resources

In the three surveys conducted in March 2021, February 2023, and November 2023, we performed a comprehensive evaluation of fish resources by integrating biological trawl and acoustic survey data. These surveys revealed seasonal variations in fish length distributions and the characteristics of their target strength (TS) in acoustic monitoring, providing a basis for resource assessment through acoustic methods [54,55,56]. Target strength is a key parameter in acoustic surveys used to estimate fish volume and biomass, primarily influenced by fish length, body shape, and internal gas content (e.g., swim bladders) [57,58,59,60,61]. Acoustic surveys offer broader spatial coverage and greater efficiency compared to traditional trawl surveys, especially when assessing fish abundance over large water areas. These advantages are particularly evident in wide-ranging aquatic environments [62]. In this study, the acoustic transect data covered a large marine area, and the analysis of echo integration values and target strength yielded fish density and biomass estimates for each survey station. A comparison of the fish density and biomass distribution maps derived from trawl surveys and acoustic transect data revealed consistent patterns of resource concentration. Both methods indicated that areas with higher fish density were primarily located along the coasts of the mainland and islands. This concentration can likely be attributed to the higher primary productivity in coastal areas, which provides ample food resources. Additionally, the shallower depths in these regions make them ideal spawning and nursery grounds for fish [63,64].

Although both trawl and acoustic surveys have their advantages and limitations, combining these two methods can maximize the accuracy of fish abundance assessments [65]. Trawl surveys involve the direct capture of fish, providing valuable biological information, such as species composition, body length, weight, sex, and age, making them essential for assessing fish community structure and health [66]. Additionally, smaller or fast-swimming species may escape the net, leading to inaccuracies in abundance estimates [66,67]. In contrast, acoustic technology can distinguish fish of varying sizes using target strength and offers detailed vertical distribution data, making it particularly useful for large-scale water assessments. It also operates more efficiently than traditional trawl surveys [68]. However, acoustic surveys estimate fish size and abundance based solely on target strength and cannot directly identify species. Therefore, biological surveys, such as trawl sampling, are needed to validate and calibrate species composition data [69]. By integrating these two methods, acoustic surveys can provide preliminary assessments over large areas, while trawl surveys allow for more refined analysis and data calibration, especially for species identification and benthic fish evaluation [65,70,71]. These technologies have also tracked lobster movements at depths of up to 400 m [72] and identified the echo characteristics of jellyfish and krill, proving effective for analyzing distribution densities in sensitive marine areas [73,74]. This integrated approach not only improves the accuracy of fish abundance assessments but also provides robust data for fishery management, ecological conservation, and intake safety assessments for nuclear power plants.

Considering the economic aspect, traditional trawl surveys are time-consuming and costly. In our study, a large amount of manpower was required for setting up the trawl nets, operating the vessels, and processing the biological samples each time a trawl survey was carried out. The cost of vessel operation, including fuel expenses, crew salaries, and equipment maintenance, was approximately 8000 RMB per survey. In addition, the trawl gear suffered from wear and tear and needed to be replaced regularly, which also increased the total cost. In contrast, although acoustic surveys required an initial investment in equipment such as the BioSonics DT-X scientific echosounder (USA) and related software, the cost per survey was lower in the long run. The initial purchase cost of the acoustic equipment was about 100,000 RMB. However, considering the long-term and multiple surveys carried out over time, the average cost per survey became relatively lower. This cost-effectiveness, combined with its high efficiency in large-scale assessments, makes acoustic technology a more advantageous option in terms of economic evaluation for long-term fish resource monitoring. Acoustic monitoring can be integrated into the real-time early warning system of nuclear power plants, and the risk of biological blockage can be reduced by dynamically adjusting the water intake strategies. Moreover, this method can be applied to monitor fish populations in commercial fishing areas [75,76]. By regularly conducting surveys, fishery managers can accurately estimate the biomass and distribution of fish, which is crucial for setting sustainable fishing quotas. For example, in large-scale fishing grounds, acoustic surveys can quickly identify areas with high fish densities, and then trawl surveys can be used to sample and analyze the species composition.

### 4.4. Error Sources of Trawl and Underwater Acoustic Techniques

There are multiple sources of error in trawl technology during fish resource surveys. The escape rate is an important issue, as small or fast-swimming fish can easily escape from the nets. Studies have shown that different mesh sizes have a significant impact on the escape rate [77,78]. Although a small mesh size can reduce the escape of small fish, it will increase the resistance of the nets and affect the trawl efficiency [78]. In the swept area method used in this study, the determination of the capture efficiency (*q* value) is crucial. The capture efficiency of different fish species is determined through experiments to minimize errors caused by the selectivity of the nets as much as possible. However, in order to quantify the escape rate more accurately, it is recommended that subsequent studies supplement comparative experiments with different meshes to obtain more accurate escape rate data. Regarding trawl errors, correction methods are mainly achieved through experiments and models. In terms of the experiments, comparative experiments with different mesh sizes can be carried out, the escape situations of various fish under different meshes can be recorded, and a relationship model between the escape rate, mesh size, fish species, and fish size can be established so as to correct the capture data.

The sources of error in acoustic technology are rather complex. The dependence of target strength (TS) on fish body length is one of the main error factors [32,33]. Although Equation (5) describes the relationship between TS and body length, differences in fish body morphology, such as the shape and size of the swim bladder, can cause deviations between the actual TS and the theoretical values [58,61]. In addition, environmental noise interference, scattering and absorption in the sound wave propagation path, and equipment calibration deviations can also affect the results of acoustic surveys [79]. Environmental noise may mask the echo signals of fish, and the scattering and absorption of sound waves during propagation can lead to signal attenuation and reduce the detection accuracy; equipment calibration deviations may make the measured TS values inaccurate. To correct the errors of acoustic technology, multiple methods can be adopted. In terms of TS calibration, using standard targets for calibration is a common means. By comparing the echoes with those of standard targets, the equipment can be calibrated. Meanwhile, combining on-site trawl data for verification and conducting a comparative analysis of the body length and species of fish captured by trawl with the TS values measured acoustically can further improve the accuracy of TS values.

### 4.5. Impact of the Research Results on the Safety of the Cooling Water Intake System of Nuclear Power Plants

This study conducted a systematic analysis of the spatiotemporal distribution as well as the variations in biomass of fish resources within the water intake areas of nuclear power plants. When considering the spatiotemporal distribution of species, notable differences were observed in the quantity of fish species, resource density, and biomass density across different seasons and sampling stations. For instance, the individual density of fish reached its peak during the survey conducted in February 2023, whereas the biomass density attained its highest level in November 2023. These variations mirror the seasonal behaviors of fish, including spawning, migration, and foraging, as documented in previous studies [80,81,82]. During the operation of the water intake process in nuclear power plants, these behavioral patterns of fish may result in a substantial congregation of fish in the vicinity of the water intake areas at specific times. In the absence of timely monitoring and early warning mechanisms, there is a high probability that such congregations could lead to the blockage of the water intake system, thereby posing a threat to the safe operation of the nuclear power plants.

Through an in-depth analysis of dominant species, it has been revealed that the dominant species differ across various seasons. Specifically, in February 2023, species such as *C. lucidus* prevailed, whereas in November 2023, species like *H. nehereus* became predominant. The alterations in these dominant species are closely intertwined with environmental factors and further exert a significant influence on the distribution and aggregation patterns of fish within the water intake areas. Certain species, exemplified by *C. lucidus*, which possess strong adaptability and high reproductive capabilities, are capable of maintaining relatively high population numbers throughout different seasons. This phenomenon consequently heightens the risk of blockage within the water intake system. Hence, precisely grasping the dynamic variations of dominant species is of paramount importance for predicting and forestalling the biological blockage issues that may afflict the water intake system.

The outcomes of acoustic assessments demonstrate that acoustic technology is not only highly efficient but also remarkably reliable in monitoring fish resources. It enables the swift acquisition of information regarding the distribution and quantity of fish across extensive water areas. When combined with trawl survey data, a more precise evaluation of fish resources can be achieved. This holds crucial guiding significance for nuclear power plants in formulating scientific water intake strategies as well as implementing effective biological prevention and control measures. Via acoustic monitoring, should an abnormal increase in the density of fish resources be detected near the water intake area, timely adjustments to the water intake mode can be made, or corresponding deterrent measures can be adopted promptly. By doing so, the likelihood of fish entering the water intake system can be effectively reduced, thereby ensuring the safety of the water intake system.

Moreover, this study has additionally discovered a significant negative correlation between water depth and the density of fish resources. It has been observed that fish resources are predominantly concentrated in shallower regions. Given that the water intake areas of nuclear power plants are typically situated in nearshore shallow areas, this implies that fish aggregation is more prone to occur in these specific zones. Consequently, when engaged in the design and management of the water intake system, this factor must be thoroughly considered. Strengthened efforts should be directed toward the monitoring, prevention, and control of fish in shallow areas. For instance, drum filters and wedge wire screens can be installed. Drum filters are effective in removing large-sized marine organisms, while wedge wire screens can filter out smaller particles and organisms.

## 5. Conclusions

This study systematically analyzed the spatiotemporal distribution and biomass variation of fish resources in the water intake zone of a nuclear power plant by integrating acoustic surveys and biological trawling. The results revealed significant seasonal and spatial differences in species composition and density, reflecting changes in ecological niches and population dynamics. The seasonal variation of dominant species, such as *C. lucidus* and *D. volitans*, demonstrated the adaptability and ecological strategies of fish to environmental changes. The consistency between acoustic and traditional trawl survey results further validated the reliability and efficiency of acoustic technology in fish resource assessments. Acoustic methods excel in providing broad spatial coverage and fine-scale vertical resolution, making them particularly effective for monitoring resource density across large marine areas. These surveys can quickly and accurately capture the vertical distribution of fish populations across different water layers. Meanwhile, trawl surveys, by directly capturing specimens, offer critical biological information such as species composition, length, and weight, providing essential reference points for calibrating and validating acoustic data. The combined use of both methods enhanced the comprehensiveness and precision of fish resource assessments. Future research will focus on optimizing acoustic survey techniques, expanding survey areas, and integrating long-term environmental monitoring to uncover the mechanisms driving changes in fish resources. In sensitive areas, like the water intake zone of nuclear power plants, environmental factors, such as temperature, salinity, nutrient concentrations, and ocean currents, may have profound impacts on fish populations. High-frequency monitoring and multivariate analysis models will be employed to explore the complex relationships between environmental changes and fish resource dynamics. The use of acoustic surveys based on unmanned platforms will be a key focus in future research, aiming to improve monitoring efficiency and data precision. Moreover, future research needs to address gaps in the development of unmanned platform-based acoustic survey technologies, high-performance acoustic equipment, and the construction of a multi-species acoustic scattering target library in China [83].

The findings of this study provide scientific evidence to support the ecological safety management of the nuclear power plant’s cooling water intake and offer valuable insights for the sustainable use of regional marine resources. By precisely tracking the dynamic changes in fish resources in the intake area, we can ensure the safe operation of the plant while developing scientifically sound fishery management strategies to minimize ecological impacts. Ultimately, this dual approach aims to achieve a win–win outcome of ecological conservation and resource utilization.

## Figures and Tables

**Table 1:** Information on the trawl survey of fishery resources in the nuclear power plant water intake area.

Time	Net Set	Longitude of Net Setting	Latitude of Net Setting	Longitude of Net Hauling	Latitude of Net Hauling	Average Speed (n mile/h)	Towing Duration (h)
March 2021	1	120°18.430′	27°04.760′	120°20.334′	27°04.455′	3.7	0.50
	2	120°20.496′	27°03.182′	120°18.612′	27°03.293′	3.3	0.50
	3	120°18.549′	27°03.549′	120°18.465′	27°03.101′	4.0	0.50
	4	120°23.100′	27°01.588′	120°21.326′	27°01.605′	3.2	0.50
	5	120°16.779′	27°01.362′	120°18.589′	27°00.704′	3.4	0.50
	6	120°19.995′	26°58.917′	120°17.589′	26°57.737′	4.0	0.50
	7	120°16.877′	26°59.400′	120°16.548′	27°01.190′	3.7	0.50
February 2023	1	120°17.421′	27°03.724′	120°19.352′	27°03.701′	3.5	0.55
	2	120°19.523′	27°03.709′	120°21.363′	27°03.168′	3.6	0.58
	3	120°25.866′	27°04.610′	120°26.144′	27°02.987′	3.5	0.52
	4	120°21.695′	27°01.999′	120°19.179′	27°02.027′	3.0	0.78
	5	120°16.926′	27°01.182′	120°19.352′	27°00.462′	3.4	0.83
	6	120°24.612′	26°59.174′	120°24.160′	26°55.924′	3.8	0.85
	7	120°19.708′	26°54.562′	120°17.521′	26°54.793′	3.1	0.70
November 2023	1	120°17.912′	27°03.676′	120°19.679′	27°02.831′	3.3	0.50
	2	120°20.027′	27°02.766′	120°22.483′	27°02.223′	3.6	0.50
	3	120°26.268′	27°04.467′	120°24.116′	27°03.511′	4.5	0.50
	4	120°26.110′	27°02.234′	120°26.513′	27°04.242′	4.7	0.50
	5	120°19.315′	27°01.212′	120°17.805′	27°01.748′	4.8	0.50
	6	120°18.914′	27°00.980′	120°21.211′	27°00.849′	5.0	0.50
	7	120°27.054′	26°59.532′	120°26.143′	26°58.045′	2.5	0.50
	8	120°25.548′	26°57.180′	120°24.559′	26°55.752′	3.3	0.50
	9	120°20.035′	26°54.420′	120°17.290′	26°54.835′	3.6	0.50

**Table 2:** Coefficient *b*_20_ and fishing gear catch rate *q* for the captured fish in the surveyed area [30,31,32]. (*b*_20_ is the target strength parameter).

Species	*b*_20_	*q*	Species	*b*_20_	*q*
*Arius sinensis*	−66.1	0.8	*Cynoglossus lighti*	−71.9	0.8
*Thryssa mystax*	−72.5	0.3	*Liza haematocheila*	−72.5	0.3
*Chaeturichthys hexanema*	−71.9	0.8	*Nibea albiflora*	−68.0	0.5
*Coilia mystus*	−72.5	0.3	*Sillago sihama*	−72.5	0.3
*Coilia nasus*	−72.5	0.3	*Cociella punctata*	−68.0	0.5
*Larimichthys crocea*	−68.0	0.5	*Tridentiger barbatus*	−71.9	0.8
*Trypauchen vagina*	−71.9	0.8	*Johnius grypotus*	−68.0	0.5
*Eupleurogrammus muticus*	−66.1	0.5	*Miichthys miiuy*	−68.0	0.5
*Chrysochir aureus*	−68.0	0.5	*Larimichthys polyactis*	−68.0	0.5
*Collichthys lucidus*	−68.0	0.5	*Solea ovata*	−71.9	0.8
*Chaemrichthys stigmatias*	−71.9	0.8	*Sebastiscus marmoratus*	−67.7	0.3
*Cynoglossus joyneri*	−71.9	0.8	*Lateolabrax japonicus*	−72.5	0.5
*Odontamblyopus rubicundus*	−71.9	0.8	*Acanthogobius ommaturus*	−71.9	0.3
*Sebastiscus marmoratus*	−67.7	0.8	*Dactylopterus volitans*	−72.5	0.5
*Thryssa kammalensis*	−72.5	0.8	*Hemitrygon akajei*	−71.9	0.8
*Harpadon nehereus*	−70.6	0.3	*Callionymus beniteguri*	−69.5	0.5

**Table 3:** Number of swimming animal and fish species and fish resource density and biomass density from the three surveys.

Survey Cruise	Diversity of Swimming Species	Fish Species Count	Fish Percentage (%)	Average Resource Density (ind./km²)	Average Resource Weight Density (kg/km²)
March 2021	54	26	48.15	6855	107.63
February 2023	43	29	67.44	17,267	19.02
November 2023	45	27	60.00	16,233	198.66

**Table 4:** ANOVA of resource density and weight density among survey stations.

Survey Cruise	Density	F-Value	*p*-Value	Minimum Value	Maximum Value
March 2021	Resource density	2.567	<0.05	2638 ind./km^2^	10,256 ind./km^2^
	Weight density	5.773	<0.01	43.347 kg/km^2^	625.060 kg/km^2^
February 2023	Resource density	2.661	<0.05	4556 ind./km^2^	17,267 ind./km^2^
	Weight density	2.070	<0.05	3.671 kg/km^2^	46.576 kg/km^2^
November 2023	Resource density	9.378	<0.05	2343 ind./km^2^	27,979 ind./km^2^
	Weight density	2.121	<0.05	44.704 kg/km^2^	519.171 kg/km^2^

**Table 5:** Relationship between fish target strength and body length in the acoustic survey of the water intake area of a nuclear power plant.

Target Strength (dB)	March 2021		February 2023		November 2023	
	Body Length (cm)	Percent (%)	Body Length (cm)	Percent (%)	Body Length (cm)	Percent (%)
−90~−85	0.11~0.19	0.73	0.10~0.14	0.20	0.12~0.22	1.06
−85~−80	0.19~0.34	1.00	0.14~0.25	16.01	0.22~0.38	18.43
−80~−75	0.34~0.60	4.47	0.25~0.44	39.98	0.38~0.68	31.52
−75~−70	0.60~1.06	61.42	0.44~0.78	17.52	0.68~1.21	24.26
−70~−65	1.06~1.89	26.26	0.78~1.39	11.88	1.21~2.15	14.69
−65~−60	1.89~3.37	5.08	1.39~2.47	9.26	2.15~3.82	6.47
−60~−55	3.37~5.98	0.75	2.47~4.39	4.03	3.82~6.80	2.16
−55~−50	5.98~10.64	0.11	4.39~7.81	0.76	6.80~12.09	0.96
−50~−45	10.64~18.92	0.05	7.81~13.88	0.20	12.09~21.50	0.34
−45~−40	18.92~33.65	0.08	13.88~24.69	0.10	21.50~38.24	0.09
−40~−35	33.65~59.84	0.05	24.69~43.90	0.05	38.24~68.00	0.01
−35~−30	59.84~106.41	0.01	43.90~133.40	0.00	68.00~120.92	0.00

**Table 6:** Fish echo integration value and target strength from an acoustic transect survey in the water intake area.

Survey Cruise	Integration Value (dB)	Maximum Target Strength (dB)	Minimum Target Strength (dB)	Average Target Strength (dB)	Average Depth (m)
March 2021	95,744.85	−42.42	−57.90	−47.90 ± 13.59	19.10 ± 16.32
February 2023	30,722.18	−40.01	−54.72	−46.11 ± 12.59	8.05 ± 3.95
November 2023	512,361.35	−21.17	−80.95	−69.35 ± 6.11	6.70 ± 3.20

## Data Availability

The original contributions presented in this study are included in the article. Further inquiries can be directed to the corresponding authors.
